# Adapting to New Regulations on Psychiatric Seclusion: Organizational and Clinical Impacts in an Adult Psychiatry Unit

**DOI:** 10.1192/j.eurpsy.2026.11298

**Published:** 2026-07-31

**Authors:** S. Boudour, A. Amina

**Affiliations:** EPS Barthelemy Durand, Etampes, France

## Abstract

**Introduction:**

Recent regulatory changes governing seclusion and restraint in psychiatry now strictly prohibit the use of seclusion outside of a dedicated and compliant room. Fully enforced since August 2025, this requirement represents a major structural shift for psychiatric services, necessitating rapid organizational and architectural adaptation.

**Objectives:**

To describe the adaptation process of an adult psychiatric service to this new regulation, assess its impact on clinical and organizational practices, and identify challenges encountered as well as facilitating factors for compliance.

**Methods:**

A retrospective descriptive study analyzing seclusion and restraint events between January and December 2025. The analysis compares the period preceding the prohibition of seclusion in non-dedicated rooms (up to August 2025) and the period following full compliance implementation. Data were collected from regulatory registers, institutional meetings, and qualitative feedback from clinical teams.

**Results:**

The implementation of a dedicated and compliant seclusion room significantly transformed care pathways for patients exhibiting acute behavioral disturbances. Early observations indicate a radical shift in the conceptualization of the patient care pathway, with a readjustment of initial management strategies, reflecting a rapid evolution of practices and strengthened staff engagement. Eliminating seclusion in non-dedicated rooms required internal reorganization, updated protocols, and ongoing resource adjustments despite initial architectural and logistical constraints.

**Conclusions:**

Compliance with the new regulatory framework for psychiatric seclusion represents a major institutional transition. It has driven the reconfiguration of clinical practices, the development of alternative strategies, and increased organizational support needs. Future improvement efforts should focus on continuous staff training, optimization of secure care spaces, and resource allocation to ensure safe, ethical, and legally compliant patient care.

**Disclosure of Interest:**

None Declared

